# Brain Abnormalities During Self-Referential Task in Subthreshold Depression: A Near-Infrared Spectroscopy Study

**DOI:** 10.7759/cureus.75351

**Published:** 2024-12-08

**Authors:** Waka Nakano, Satoshi Yokoyama, Hiroshi Sato

**Affiliations:** 1 Division of Humanities and Social Sciences, Graduate School of Humanities and Social Sciences, Hiroshima University, Higashihiroshima, JPN; 2 Department of Humanities, Faculty of Humanities, Niigata University, Niigata, JPN; 3 Department of Integrated Psychological Sciences, School of Humanities, Kwansei Gakuin University, Nishinomiya, JPN

**Keywords:** bayesian statistics, depression, near-infrared spectroscopy, prefrontal cortex, subthreshold depression, university student

## Abstract

Background and aim

Subthreshold depression is a potential risk factor for major depressive disorder. Although the neurobiological mechanism underlying major depressive disorder is well-established, the mechanism underlying subthreshold depression has not yet been fully elucidated. We investigated the characteristics of brain abnormalities in participants with subthreshold depression using near-infrared spectroscopy (NIRS) owing to its portability.

Methods

A total of 53 college students were recruited, and all participants performed three tasks: self-referential task (SRT), verbal fluency task (VFT), and category fluency task (CFT). Hemodynamic changes in the prefrontal cortex (PFC) during the tasks were also measured using NIRS. A generalized linear model was employed for the NIRS data analysis. Subsequently, we evaluated the relationship between depressive severity and NIRS data during task performance.

Results

Our analysis revealed a positive correlation between hemodynamic changes in the right PFC during the SRT and depression severity (SRTL_coeff: 0.12), suggesting that increased activation in this region may be associated with higher levels of depressive severity. In contrast, hemodynamic changes in the left PFC during the SRT did not significantly influence the severity of depression. Additionally, hemodynamic changes during the VFT and CFT did not significantly influence the severity of depression.

Conclusions

Hyperactivation of the right PFC, which is a characteristic of subthreshold depression, may cause a negative bias, leading to high sensitivity to negative stimuli. These results provide novel insights into the neural mechanism of subthreshold depression and highlight the utility of NIRS for evaluating brain function related to negative bias in the right PFC.

## Introduction

Subthreshold depression is characterized by depressive symptoms that are not diagnosed with major depressive disorder (MDD) [1]. The diagnosis of MDD requires other symptoms, such as weight changes, sleep difficulties, psychomotor agitation or retardation, fatigue or loss of energy, diminished ability to concentrate, feeling excessive guilt, and suicidality, as well as depressed mood or anhedonia, which is the loss of interest or pleasure [2]. Compared with MDD, subthreshold depression is often overlooked because it does not have symptoms other than depressive symptoms. Furthermore, little attention has been paid to subthreshold depression in the academic community [3]. However, some studies have suggested that subthreshold depression is a possible precursor to MDD [4-6]. Therefore, studying subthreshold depression is essential for preventing MDD.

Neuroimaging techniques, including functional magnetic resonance imaging (fMRI) and near-infrared spectroscopy (NIRS), have been used to elucidate the neurobiological mechanisms underlying subthreshold depression. In particular, considering its advantages such as complete non-invasiveness, portability, and relatively low cost compared with fMRI, NIRS has been adopted to measure brain activity in cerebral blood flow [7]. NIRS studies suggest that subthreshold depression results in functional brain abnormalities in the prefrontal cortex (PFC) [8]. In particular, the left PFC is a key region in subthreshold depression [9,10].

Previous studies on subthreshold depression used the verbal fluency task (VFT) and category fluency task (CFT) as activation tasks [9,10]. Individuals were asked to generate as many different words as possible that followed a certain rule, which was specifying an initial letter in the VFT or a conceptual category in the CFT, within a limited timeframe. According to the review, both tasks seem to reflect brain activity in the left PFC, which governs cognitive functions, such as executive control, lexical access ability, and information processing speed [11]. Although previous studies have suggested that participants with subthreshold depression exhibit hypoactivity in the left PFC during tasks [12-14], the exact mechanisms by which brain abnormalities in the left PFC cause depressive symptoms remain unclear.

In contrast to the VFT and CFT, the self-referential task (SRT) during NIRS examinations can reveal brain abnormalities related to emotional-cognitive function, which may cause depressive symptoms. In the SRT, participants were instructed to judge whether each trait word described themselves [14]. Therefore, it is easy to explain the relationship between depressive symptoms and brain abnormalities observed during the SRT. Prior studies have identified brain abnormalities in the PFC during SRT as key to subthreshold depression [15,16]. While the SRT is useful as an fMRI activation task for evaluating brain activity indicative of depressive symptoms, its effectiveness as a NIRS activation task remains unknown.

This study investigated subthreshold depression-related brain activation during the SRT using NIRS. By comparing NIRS and fMRI, a previous study found that NIRS signals were highly correlated with fMRI measurements [15]. A previous fMRI study reported that participants with subthreshold depression exhibited hyperactivity in the PFC during the SRT [16,17]. Moreover, a systematic review that established the capacity of NIRS to detect brain activation changes suggested that it could detect brain activation changes involved in emotional processing [18]. Based on these results, we hypothesized that the NIRS data of participants with subthreshold depression would show higher activation of negative words during the SRT than the data of healthy participants.

Hypothesis: The NIRS data of participants with subthreshold depression would show higher activation of negative words during the SRT than the data of healthy participants.

## Materials and methods

We conducted an NIRS study from October to November 2023 at a private university in Japan, Kwansei Gakuin University. This study was approved by the Kwansei Gakuin University Institutional Review Board for Behavioral Research with Human Participants (2023-24).

Participants

All participants were recruited from among the students of a private university. To recruit inventory participants, flyers with informed consent were displayed on campus bulletin boards and provided to participants attending classes on campus. The Japanese version of the Beck Depression Inventory-II (BDI-II) was used to assess depressive symptoms [19]. The reliability and validity of the Japanese version of the BDI-II were confirmed [20]. Each question is analyzed on a four-point scale, with higher scores serving as higher indicators of depression. According to the prior study [3], participants were categorized into two types: healthy (BDI-II score < 10) and subthreshold depression (10 ≤ BDI-II score). Participants were randomly selected from an inventory of participants of each type (healthy and subthreshold depression) using computer-generated random number sequences. All the participants provided written informed consent before participating in the study. The inclusion criteria were age ≥ 18 and ≤ 23 years, any gender, current university enrollment, and voluntary participation in the study. The exclusion criteria comprised major depressive episodes within the past year, lifetime history of bipolar disorder or psychiatric treatment (medications or psychotherapy), and the acute risk of suicide attempt. The sample size was estimated by setting a medium effect size, α = 0.05, and power = 0.80.

NIRS

We evaluated oxygenation changes in the PFC using mobile HOT-2000 (NeU, Tokyo, Japan). Mobile NIRS has been used in various studies, including those exploring the relationship between cognitive training, brain activity monitoring, and psychophysical evaluation of driver states [21]. The device measured the relative changes in total hemoglobin (total-Hb) concentration at a wavelength of 810 nm and sampling rates of 100 ms. The left probes were positioned at Fp1 or F7 and the right probes were positioned at Fp2 or F8, based on the International 10-20 system.

Task

SRT

**Figure 1:**
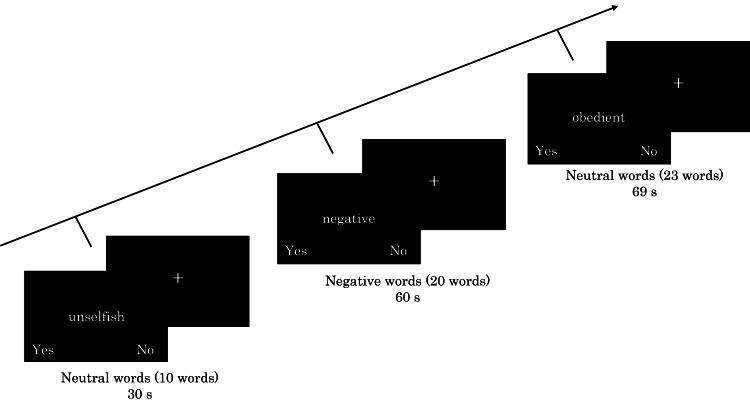
Example of experimental presentation during SRT. SRT, self-referential task

SRT was performed with the same procedure including trait words as the previous study [22]. Participants were instructed to judge whether each trait word described them. Participants made a “Yes” or “No” response by pressing a button to indicate their decision. Trait words were divided into three groups: neutral words (10 words), negative words (20 words), and neutral words (23 words). A previous study have demonstrated that there was no significant difference in character count across groups [22]. Trait words were presented randomly within the groups and centrally on the screen for 2.5 seconds. Then, a 0.5-second interval followed the next trait word. Both response keys and reaction times were recorded. Figure 1 shows an example of the experimental procedure.

VFT and CFT

We performed the VFT and CFT as positive controls to help interpret brain activity during the SRT. Following the paradigm used in a previous study, the procedure consisted of a 30-second pre-task period, a 60-second task period, and a 70-second post-task period [23]. During the pre- and post-task periods, participants were asked to say “a, i, u, e, o” as a baseline task. During the task period in VFT, participants were instructed to generate as many words as possible, beginning with “a,” “ki,” and “ha,” which are some of the 50-character Japanese hiragana syllabaries, for the 20 s per syllable type. During the task period in CFT, participants were instructed to generate as many words as possible, belonging to the semantic categories of “drink,” “vegetable,” and “sports,” for the 20-second per category. Before the trials, participants were asked to practice VFT with different letters from “a,” “ki,” and “ha.”

Prior to task administration, a one-minute calibration was performed. During this period, participants were instructed to focus on the display screen. Subsequently, participants were asked to minimize body movements, maintain attention on the task screen, and generate as many responses as possible, even when experiencing difficulty with word generation. After completing the three tasks (SRT, VFT, and CFT), the participants removed the NIRS devices and answered the desirability and accessibility scales (7 points) for each word in the SRT.

Analysis

Behavioral Data

To examine whether behavioral data (performance in the VFT and CFT, response rate, response time, desirability, and accessibility) differ across groups, a multivariate analysis of variance (MANOVA) was conducted, with group as the independent variable and behavioral measures as dependent variables. Wilks’ lambda was employed as the multivariate test statistic to evaluate the overall effect of the independent variables on the dependent variables in MANOVA. Wilks’ lambda helps to assess multivariate effects by providing a measure of the variance explained by the model compared to the unexplained variance. The threshold for statistical significance was set at *p* < 0.05. Prior to conducting the MANOVA, we performed the Shapiro-Wilk test and to assess data normality and Box’s M test to evaluate the homogeneity of variance-covariance matrices. It should be noted that the analysis of behavioral data was positioned as a secondary indicator.

NIRS Data

NIRS signals were processed according to a previously described method of a prior study [22]. The increased hemoglobin (Hb) during the task period is assumed to return to baseline.within 50 seconds. Therefore, the end 10 seconds of the pre-task period and.5 seconds from 50 seconds to 55 seconds of the post-task period were.adjusted as the baseline. Median filtering techniques and moving average factors were used to remove the instrument noise and short-term motion artifacts, respectively. We then calculated the mean total-Hb change using a real-time scalp signal separating (RT-SSS) algorithm [24]. Increased mean total-Hb changes indicate enhanced regional cerebral blood flow, suggesting higher brain activation. However, decreased total-Hb levels were associated with reduced cerebral blood flow, indicating lower brain activation. A MANOVA was performed to compare the mean total-Hb change in each channel activated during the tasks between the groups. The significance level was set at *p* < 0.05. SPSS Version 23 (IBM Corp., Armonk, NY) was used for the analysis.

Generalized Linear Modeling and Parameter Estimation

We used a generalized linear model (GLM) to investigate the relationship between subthreshold depression and brain activation. We hypothesized that participants with subthreshold depression would show greater total-Hb changes than healthy participants during SRT. In other words, we anticipated that subthreshold depression would be associated with total-Hb changes during tasks. We constructed a model in which depression scores were influenced by total-Hb changes in the left and right PFC during the tasks (VFT, CFT, and SRT). We included total-Hb changes during the VFT and CFT in the model because these tasks served as positive controls, which helped interpret the results of the SRT. Our model can be described as follows:

$\small \left ( BDI_{i} \right ) = Poisson\left ( \beta _{0}+ \beta _{1}\left ( VFTL \right )+ \beta _{2}\left ( VFTR \right )+ \beta _{3}\left ( CFTL \right )+ \beta _{4}\left ( CFTR \right )+ \beta _{5}\left ( SRTL \right )+ \beta _{6} \left ( SRTR \right )+ \varepsilon_{i}\right)$

*BDI_i_* described the depressive score. *i and β_0_* described participants and the intercept, respectively. *β_1_* and *β_2_* described the regression coefficients for total-Hb changes during VFT in the left and the right PFC, respectively. *β_3_* and *β_4_* described the regression coefficients for total-Hb changes during CFT in the left and the right PFC, respectively.*β_5_* and *β_6_* described the regression coefficients for total-Hb changes during SRT in the left and the right PFC, respectively. *ε_i_* described the residual. 

We employed Bayesian parameter estimation using our model. Bayesian parameter estimation uses a numerical algorithm to obtain posterior samples of parameter estimation [25]. The detailed settings of the algorithm were as follows: the probability distribution was a Poisson distribution, all iterations were set to 30,000, the burn-in samples were set to 1,000, and the number of chains was set to 4. The probability was transformed into a z-score. These settings are commonly used to ensure adequate parameter estimation. Before interpreting the results, we ensured that the R-hat for all the parameters was equal to 1.0, which is a strong indicator of convergence. Analyses were performed using the R statistical software (version 4.2.1, R Foundation for Statistical Computing, Vienna, Austria).

## Results

A total of 244 participants (49 males, 192 females, 3 not reported; mean age = 19.56 years, SD = 1.53 years, 18-23 years) completed the inventory. The total number of experimental participants was 57 (4 males, 51 females, 2 not reported; mean age = 19.75 years, SD = 1.41 years, 18-23 years).Participants were excluded if their data were unusable. The final sample consisted of 27 healthy participants (2 males, 25 females; mean age = 20.11 years, SD = 1.37 years, 18-22 years), 26 with subthreshold depression (2 males, 22 females, 2 not reported; mean age = 19.32 years, SD = 1.21 years, 18-23 years).

Behavioral data

Table 1 describes the results of the MANOVA with the six test measurements as dependent variables (performance on the VFT and CFT, reaction rate, reaction time, desirability, and accessibility scores). There were no significant group differences in any of the dependent variables or groups (F (6, 42) = 2.22, p = 0.06: Wilks’ lambda).

**Table 1:** Behavioral data for healthy and subthreshold depression. VFT indicates the number of words as performance in VFT, CFT indicates the number of words as performance in CFT, desirability indicates the desirability rating, accessibility indicates the accessibility rating, reaction rate indicates the reaction rating in SRT, reaction time indicates the reaction time in SRT. The value in parentheses indicates SD. CFT, category fluency task; SRT, self-referential task; VFT, verbal fluency task

	Healthy	Subthreshold depression	*F*-value	*P*-value
VFT	15.00 (3.61)	16.00 (6.21)	0.52	0.47
CFT	24.40 (5.19)	24.00 (6.67)	0.02	0.87
SRT reaction rate	6.26 (4.05)	10.1 (5.61)	7.87	0.07
SRT reaction time	1.07 (0.24)	1.04 (0.26)	0.02	0.63
Desirability	5.71 (0.68)	5.59 (1.70)	4.62	0.03
Accessibility	3.77 (1.31)	4.44 (1.55)	2.30	0.13

NIRS data

**Figure 2:**
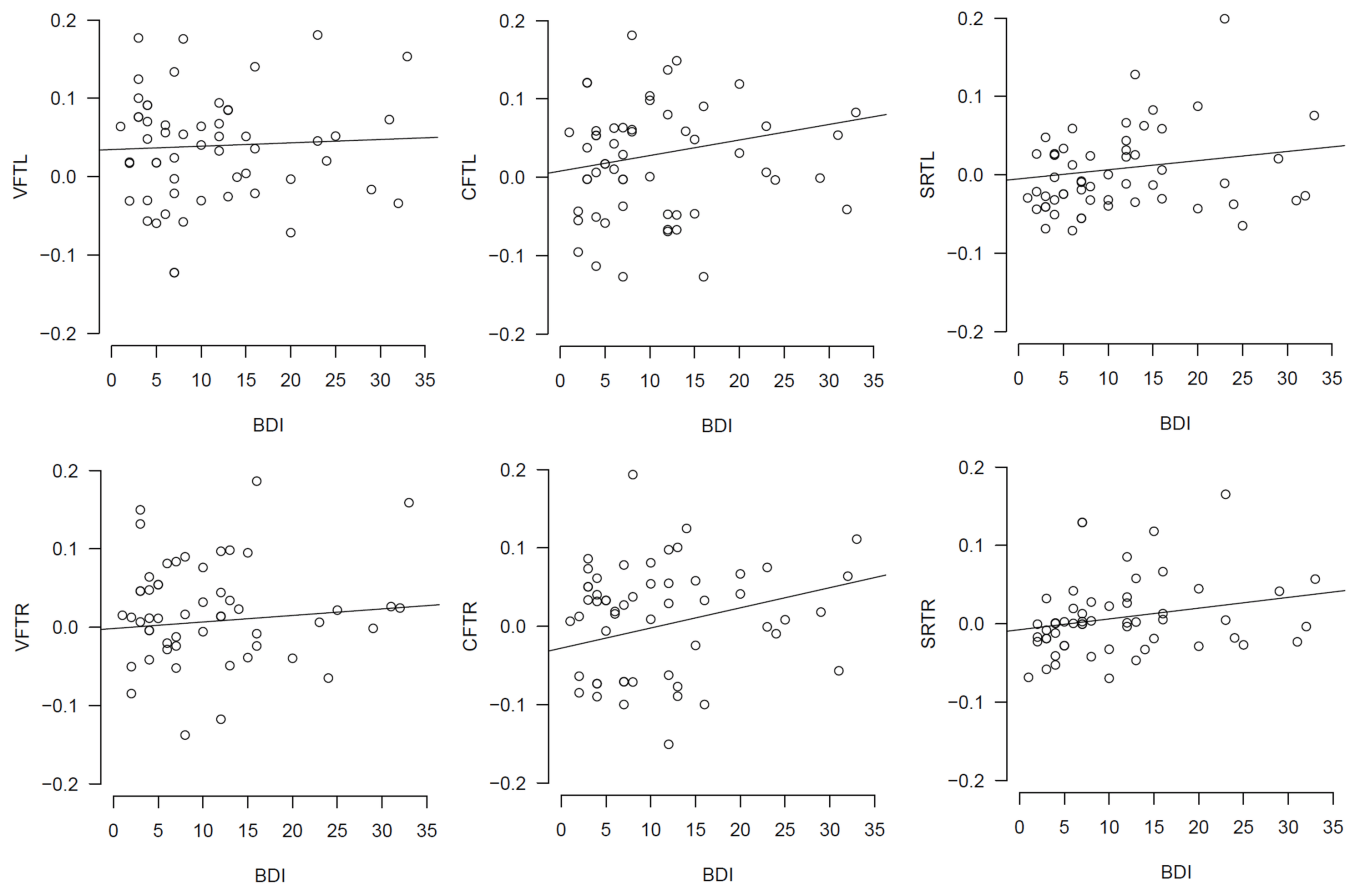
Relationship between BDI-II score and brain activation. VFTL and VFTR indicate brain activation during VFT in the left and right PFC, respectively. CFTL and CFTR indicate brain activation during CFT in the left and right PFC, respectively. SRTL and SRTR indicate brain activation during SRT in the left and right PFC, respectively. BDI-II, Beck Depression Inventory-II; CFT, category fluency task; PFC, prefrontal cortex; SRT, self-referential task; VFT, verbal fluency task

Figure 2 shows the relationship between the BDI-II score and brain activation. We conducted a GLM analysis to examine whether the BDI-II score affected the total-Hb changes during the tasks. The results of the model parameter estimations are presented in Table 2. If the 95% credibility interval of the parameters does not include 0, a significant effect can be inferred, as in classical statistical hypothesis testing. Table 2 shows a positive value for SRTR_coeff, which indicates that total-Hb changes in the right PFC during the SRT influenced the severity of depression, which implies that participants with hyperactivation in the right PFC had high depression severity. The values of the other parameters (VFTL_coeff, VFTR_coeff, CFTL_coeff, CFTR_coeff, and SRTL_coeff) were not determined. In summary, brain activity during the SRT explained depression severity, but brain activity during the VFT and CFT did not explain depression severity.

**Table 2:** Model estimation results. VFTL_coeff and VFTR_coeff indicate the regression coefficient of brain activation during VFT in the left and right PFC, respectively. CFTL_coeff and CFTR_coeff indicate the regression coefficient of brain activation during CFT in the left and right PFC, respectively. SRTL_coeff and SRTR_coeff indicate the regression coefficient of brain activation during SRT in the left and right PFC, respectively. CFT, category fluency task; PFC, prefrontal cortex; SRT, self-referential task; VFT, verbal fluency task

Parameter	Mean	SD	95% Bayesian confidence interval	*R-hat*
VFTL_coeff	0.01	0.06	-0.10 - 0.12	1.00
VFTR_coeff	0.00	0.06	-0.12 - 0.12	1.00
CFTL_coeff	-0.01	0.06	-0.12 - 0.10	1.00
CFTR_coeff	0.13	0.06	-0.00 - 0.25	1.00
SRTL_coeff	0.03	0.05	-0.06 - 0.12	1.00
SRTR_coeff	0.12	0.05	0.02 - 0.21	1.00

## Discussion

To the best of our knowledge, this study is one of the first to investigate brain activation in subthreshold depression using NIRS. Brain activity was measured during the SRT, VFT, and CFT. Our findings support the hypothesis that participants with subthreshold depression have higher activation of negative words during the SRT than healthy participants.

Our results highlighted two key points. First, brain activation in the right PFC during the SRT influences the severity of depression. Considering the function of the right PFC, hyperactivation implies a negative bias leading to high sensitivity to negative stimuli. This result is consistent with previous studies targeting subthreshold depression [22]. Second, brain activation in the left PFC during the VFT and CFT did not have a significant impact on depression severity. This raises the possibility that participants with subthreshold depression do not have abnormalities in cognitive functions dominated by the left PFC. Our result is supported by the observation of a previous study [22] but is not consistent with some studies [9,10]. Further studies are needed to clarify the details of brain abnormalities in the left PFC in individuals with subthreshold depression.

These findings have important implications for understanding the neurobiological mechanisms underlying subthreshold depression. We identified a potential role for the right PFC, but not the left PFC, as a key function of neurobiological mechanisms in subthreshold depression. The right PFC is recruited to suppress amygdala activity induced by negative emotions, such as emotional regulation and the amygdala responds to negative emotions leading to depressive symptoms [26]. A previous study found that an increase in activity in the right PFC was associated with deactivation of the amygdala during emotional cognitive tasks [27]. This implies that healthy individuals can control amygdala responses and negative emotions by recruiting the right PFC. However, it is difficult to control amygdala responses in individuals with subthreshold depression because of amygdala overactivation. Therefore, individuals with subthreshold depression require more activation in the right PFC to control amygdala responses to negative stimuli. A key strength of this study is its focus on the right PFC activity, which is closely related to the pathogenesis of subthreshold depression.

Nevertheless, several limitations should be acknowledged. Firstly, there is a possibility that participants in this study may have MDD. Since subthreshold depression is considered part of a continuum of the depression spectrum, there is no established definition that clearly distinguishes subthreshold depression from MDD. Following a prior study on subthreshold depression [3], we excluded individuals with a history of MDD or those who had sought treatment from a specialized institution. However, in Japan, the treatment rate for MDD is low, and many individuals do not seek help from specialized institutions even if they develop MDD [28]. Therefore, it cannot be ruled out that MDD was included in this study. Consequently, our findings on the neurobiological mechanisms of subthreshold depression may reflect characteristics of both subthreshold and MDD.

Secondly, differences in the NIRS indices may lead to the observation that brain activation in the left PFC does not influence the depression severity. Our NIRS measurement index differed from those used in previous studies [9,10]. There are three types of NIRS indices: oxy-Hb, deoxy-Hb, and total-Hb. The measurable indices were determined based on the number of wavelengths. Although multiple wavelengths can be used to measure the three indices, their data may be influenced by the loss of accuracy and low reliability because of pile-up effects, which complicate the determination of the count rate per wavelength [29]. However, a single wavelength has a high level of accuracy and reliability because it is not influenced by pile-up effects, but it can measure only total-Hb. In this study, we used the total-Hb measured using a single wavelength as the NIRS measurement index.

Thirdly, individual differences may have affected these results. The negative effect on physiological indicators such as the NIRS index can be caused by individual differences [30]. Considering individual differences, a generalized linear mixed model (GLMM) and Bayesian hierarchical model can estimate the parameters of brain activity. However, our study did not consider the effects of individual differences because we used a GLM. Future studies could employ a GLMM and Bayesian hierarchical model to evaluate depression using physiological indicators. Furthermore, the results may have been influenced by potential biases in gender and age distributions, as well as the sample size.

## Conclusions

In this study, participants with subthreshold depression exhibited hyperactivation of the right PFC during self-reference processing. Hyperactivation may cause a negative bias, which may be responsible for subthreshold depression. Future research should investigate whether subthreshold depression is derived from hyperactivation in the right PFC or whether the right PFC is hyperactivated as a result of subthreshold depression. Additionally, it is possible that brain abnormality in subthreshold depression could serve as a specific biomarker to distinguish between MDD and subthreshold depression.
